# Nutrition, Circadian Regulation, and the Gut–Melatonin–Visceral Adipose Tissue Axis: An Integrative Narrative Review

**DOI:** 10.3390/nu18193277

**Published:** 2026-10-05

**Authors:** Katarzyna Weronika Walkiewicz, Sylwia Dziegielewska-Gesiak, Małgorzata Muc-Wierzgoń

**Affiliations:** Department of Propedeutics of Internal Medicine and Emergency Medicine, Medical University of Silesia in Katowice, 41-902 Katowice, Poland; sgesiak@sum.edu.pl (S.D.-G.); mwierzgon@sum.edu.pl (M.M.-W.)

**Keywords:** visceral adipose tissue, melatonin, gut microbiota, circadian rhythms, chrononutrition, metabolic health, obesity, intestinal barrier

## Abstract

Visceral adipose tissue (VAT) dysfunction is a major determinant of obesity-related metabolic disorders, including insulin resistance, metabolic syndrome, cardiovascular disease, and chronic low-grade inflammation. Growing evidence indicates that VAT function is regulated by a complex interplay between nutrition, circadian rhythms, intestinal melatonin secretion, and the gut microbiota rather than by isolated metabolic pathways. The gastrointestinal tract represents an important interface between dietary exposures, microbial activity, immune signaling, and host metabolic regulation. Melatonin is a potential component of this network because it participates in circadian regulation and has been detected in gastrointestinal tissues, while experimental studies suggest that intestinal melatonin signaling may interact with gut microbial and metabolic processes. However, the cellular sources, quantitative contribution, local dynamics, and physiological significance of intestinal melatonin remain incompletely established, particularly in humans. This narrative review integrates current evidence regarding the interactions between nutrition, circadian regulation, intestinal melatonin, gut microbiota, and visceral adipose tissue. Rather than proposing a validated causal pathway, we introduce the Gut–Melatonin–Visceral Adipose Tissue (Gut–Melatonin–VAT) Axis as an integrative, hypothesis-generating conceptual framework that organizes established interactions together with candidate mechanisms requiring further experimental validation. Particular attention is given to the bidirectional relationships among dietary composition, meal timing, microbial rhythmicity, intestinal signaling, immune regulation, and VAT dysfunction. The framework may provide a basis for generating testable hypotheses concerning nutritional, chrononutritional, microbiota-targeted, and melatonin-related interventions, while recognizing that direct human evidence validating the complete pathway is currently lacking.

## 1. Introduction

Obesity is one of the most important public health challenges worldwide and is strongly associated with an increased risk of cardiovascular disease, type 2 diabetes mellitus, non-alcoholic fatty liver disease, and several cancers [1]. However, the metabolic consequences of obesity depend not only on total body fat mass but also on adipose tissue distribution. Among different adipose tissue depots, visceral adipose tissue (VAT) is considered particularly detrimental because of its strong association with insulin resistance, chronic inflammation, and cardiometabolic risk [2,3].

VAT differs from subcutaneous adipose tissue in its anatomical location, endocrine activity, vascularization, and inflammatory profile [3,4]. Excessive VAT accumulation promotes adipocyte hypertrophy, immune cell infiltration, and dysregulated secretion of adipokines and inflammatory mediators [4,5]. Consequently, VAT is increasingly recognized as a key driver of metabolic dysfunction [2,5,6].

Recent advances in microbiome research have identified the gut microbiota as an important regulator of adipose tissue biology [6]. The gut microbiota participates in digestion, energy harvesting, immune regulation, and maintenance of intestinal barrier integrity [7]. Growing evidence indicates that microbial dysbiosis contributes to VAT expansion and dysfunction through several interconnected mechanisms involving metabolic endotoxemia, immune activation, microbial metabolites, and neuroendocrine signaling [6,7,8].

The gut microbiota is not metabolically static but exhibits pronounced diurnal rhythmicity, with the composition and activity of microbial communities changing across the light–dark cycle. These microbial oscillations are closely linked to host circadian signals and are influenced by feeding behavior, dietary composition, and the timing of food intake [9,10]. Experimental studies have demonstrated that high-fat feeding can dampen the diurnal dynamics of the gut microbiome, whereas time-restricted feeding can partially restore these microbial rhythms [10]. Circadian disruption may also alter the composition and temporal organization of the intestinal microbiota, potentially contributing to disturbances in host metabolic homeostasis [9,11]. These observations suggest that nutrition and meal timing represent important environmental cues linking circadian regulation with gut microbial rhythmicity and metabolic homeostasis [10,12]. Importantly, the relationship between the circadian system and the gut microbiota appears to be bidirectional, as microbial rhythmicity may both respond to host and behavioral cues and contribute to the regulation of host metabolic processes [7,9]. Within this framework, melatonin has emerged as a potential mediator of the interaction between circadian regulation and the intestinal microbiota, with experimental studies suggesting that melatonin administration may influence microbial rhythmicity and gut microbial homeostasis, particularly under conditions of circadian or metabolic disruption [13,14,15].

Melatonin may therefore represent one candidate signaling component within this interconnected network. Beyond its well-established role in systemic circadian regulation, melatonin has been detected in gastrointestinal tissues, and experimental studies have provided evidence compatible with local intestinal synthesis or regulation [16,17]. However, the biological significance of this extra-pineal melatonin pool remains incompletely defined. In particular, evidence for the presence or local production of melatonin should be distinguished from evidence demonstrating sustained local accumulation, a quantitatively relevant contribution to circulating melatonin concentrations, or a causal effect on distant metabolic tissues such as visceral adipose tissue [13,18]. In particular, melatonin-induced changes in the gut microbiota have been associated with reduced adiposity, improved lipid metabolism, and attenuation of metabolic inflammation [18,19]. Moreover, the high membrane permeability of melatonin further emphasizes the need to distinguish local synthesis from local retention and tissue-specific exposure.

Experimental studies provide evidence that melatonin administration can influence gut microbial composition and rhythmicity and can attenuate metabolic disturbances in animal models [13,19]. In mice, circadian disruption was associated with alterations in gut microbial composition, increased intestinal lipopolysaccharide exposure, and adipose lipid dysmetabolism, whereas melatonin administration attenuated these changes [20]. Other experimental studies have shown that melatonin can restore microbial rhythmicity and reduce visceral adipose tissue accumulation in high-fat diet-fed mice [13]. However, these observations do not establish that intestinally derived melatonin is the obligatory mediator of these effects. Other pathways, including microbial metabolites, short-chain fatty acids, bile acid signaling, AMPK, sirtuins, and immune-mediated mechanisms, may operate independently or in parallel.

Accordingly, in this review, intestinal melatonin is considered a candidate component of the gut–microbiota–metabolic network rather than a formally established causal mediator. Moreover, the available evidence for nutrition and meal timing may therefore represent important environmental cues linking circadian regulation with gut microbial rhythmicity and metabolic homeostasis [10,12]. Rather than acting through a single pathway, dietary composition and the temporal organization of food intake may simultaneously influence microbial ecology, intestinal melatonin signaling, host circadian rhythms, and adipose tissue metabolism.

In this context, we propose the Gut–Melatonin–VAT Axis as an integrative, hypothesis-generating conceptual framework. The novelty of the framework does not lie in the discovery of individual pathways linking nutrition, circadian biology, gut microbiota, or adipose tissue, which have been studied extensively. Rather, the proposed framework integrates these partially overlapping pathways and places intestinal melatonin among several candidate signaling mechanisms potentially connecting nutritional and circadian cues with intestinal, immune, microbial, and visceral adipose tissue responses. The framework is intended to organize current evidence and identify testable biological relationships rather than to imply that the complete pathway has already been causally validated.

## 2. Materials and Methods

### 2.1. Literature Search and Review Design

This article was designed as a narrative review integrating current evidence on the interactions between nutrition, circadian regulation, intestinal melatonin, gut microbiota, and visceral adipose tissue (VAT). Literature searches were conducted in PubMed/MEDLINE, Scopus, and Google Scholar through August 2026. Additional relevant publications were identified through manual screening of the reference lists of key articles and reviews and meta-analyses.

The search strategy combined terms related to the major components of the proposed framework: Gut–Melatonin–VAT Axis, including “visceral adipose tissue”, “visceral fat”, “melatonin”, “intestinal melatonin”, “gut microbiota”, “gut microbiome”, “circadian rhythm”, “circadian disruption”, “chrononutrition”, “meal timing”, “time-restricted eating”, “intermittent fasting”, “short-chain fatty acids”, “intestinal barrier”, “gut permeability”, “*Akkermansia muciniphila*”, and “*Faecalibacterium prausnitzii*”. Boolean operators AND and OR were used to combine search terms.

The search focused primarily on English-language publications, with particular emphasis on recent literature. Earlier landmark studies were retained when they provided fundamental mechanistic or conceptual evidence relevant to circadian regulation, melatonin signaling, nutritional regulation of peripheral clocks, gut microbiota, or VAT biology. Reference lists of relevant publications were additionally screened to identify studies not retrieved through the database searches. Because this was a narrative review, the search was not intended to constitute a systematic review or formal meta-analysis, and no quantitative risk-of-bias synthesis was performed.

The literature was interpreted according to study type, with particular attention to the distinction between human observational studies, human intervention studies, animal experiments, and in vitro/mechanistic investigations.

### 2.2. Study Selection and Evidence Synthesis

Studies were considered relevant when they addressed at least one of the following areas: (i) nutritional regulation of circadian rhythms and peripheral clocks; (ii) melatonin synthesis, signaling, and metabolism, with particular emphasis on the gastrointestinal tract; (iii) circadian regulation of the gut microbiota; (iv) microbial metabolites, including short-chain fatty acids, and their effects on intestinal barrier function and adipose tissue metabolism; (v) interactions between gut microbiota, intestinal permeability, inflammation, and VAT; or (vi) nutritional, chrononutritional, microbiota-targeted, or melatonin-based interventions with potential relevance to metabolic health.

Experimental studies in animal models and cell culture were included when they provided mechanistic evidence relevant to the proposed Gut–Melatonin–VAT Axis. Human observational studies and clinical trials were considered particularly for evidence concerning chronotype, meal timing, dietary interventions, intermittent fasting, time-restricted eating, microbiota-targeted interventions, and metabolic outcomes. Relevant narrative reviews, systematic reviews, and meta-analyses were used to provide broader context and to identify additional primary studies.

For each relevant publication, information was extracted regarding study design, experimental model or population, dietary or metabolic exposure, circadian or melatonin-related mechanisms, microbiota-related findings, microbial metabolites, intestinal barrier function, adipose tissue outcomes, and systemic metabolic effects. Particular attention was given to mechanistic links connecting two or more components of the proposed Gut–Melatonin–VAT Axis. The evidence was synthesized narratively and organized according to the major biological components of the proposed framework. Throughout the review, evidence was explicitly classified according to experimental level, including human observational evidence, human intervention studies, animal/preclinical studies, and in vitro or mechanistic studies. Particular caution was applied when extrapolating mechanistic findings from experimental models to human physiology. Evidence supporting individual interactions was distinguished from evidence directly supporting the complete Gut–Melatonin–VAT framework.

### 2.3. Conceptual Framework

The Gut–Melatonin–VAT Axis presented in this review was developed as a conceptual framework based on the integration and narrative synthesis of the identified literature. The framework was used to illustrate the proposed interactions among dietary factors, circadian regulation, intestinal melatonin, gut microbiota, microbial metabolites, intestinal barrier function, and VAT. The proposed framework should not be interpreted as a formally validated or linear causal pathway. Rather, it integrates relationships supported to different degrees by human, animal, and in vitro evidence and identifies candidate mechanisms that may contribute to gut–adipose communication. An important principle of the framework is that the relationships among its components are considered bidirectional and context-dependent. In particular, intestinal melatonin represents a candidate signaling component rather than an established obligatory mediator. Other pathways, including short-chain fatty acids, bile acids, indole-derived metabolites, AMPK, SIRT1, inflammatory mediators, and immune signaling, may contribute independently or interactively.

The framework therefore serves primarily as a hypothesis-generating model. Its main purpose is to integrate established biological relationships into a testable conceptual structure and to identify specific questions for future experimental and clinical studies.

**Figure 1:**
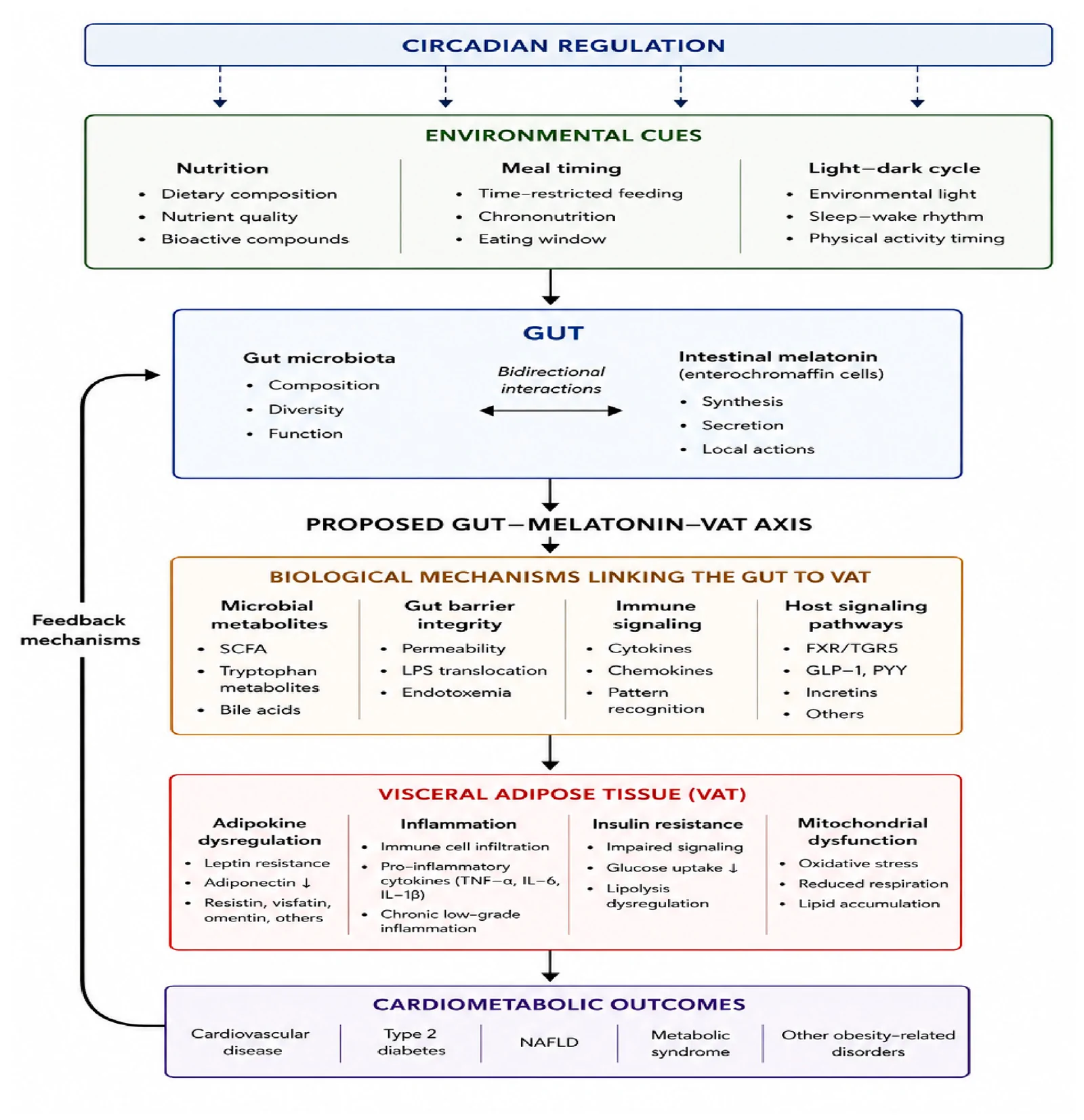
A Conceptual Model of the Gut–Melatonin–Visceral Adipose Tissue Axis (figure created by the authors based on the literature synthesis and technically generated with the assistance of an AI-based tool).

Figure 1 presents the proposed framework. The graphical representation was developed by the authors on the basis of the literature synthesis and technically assisted by an artificial intelligence-based graphical tool. AI was used only for graphical/technical assistance; the scientific concepts, literature selection, interpretation, and final content were developed and verified by the authors.

## 3. Circadian Rhythms, Melatonin, Gut Microbiota, and VAT

The gut microbiota exhibits circadian oscillations influenced by feeding behavior, sleep patterns, and host circadian regulation [10,12,14]. Disruption of these rhythms through sleep deprivation, circadian disorganization, or irregular feeding schedules may alter microbial composition and metabolic function [20]. Experimental studies further suggest that melatonin signaling may contribute to the maintenance of gut microbial rhythmicity, whereas melatonin administration may partially restore microbial alterations induced by circadian or metabolic disruption [14,15].

Melatonin has therefore emerged as a potential signaling molecule linking circadian regulation with intestinal microbial homeostasis. The relationship appears to be bidirectional: melatonin may influence microbial composition and rhythmicity, while microbial metabolism may affect intestinal melatonin-related pathways [13,16,21]. However, most mechanistic evidence originates from experimental models, and the magnitude and clinical relevance of this interaction in humans remain insufficiently defined.

The gastrointestinal tract represents an important extra-pineal site of melatonin biology. Melatonin has been detected in gastrointestinal tissues, and intestinal cells have been implicated in its local production [22,23,24]. Intestinal melatonin is structurally identical to pineal melatonin but may exert predominantly local or paracrine effects, including modulation of intestinal motility, mucosal immune responses, epithelial barrier function, and host–microbiota interactions [23,24]. Importantly, the presence of substantial amounts of melatonin in the gastrointestinal tract should not automatically be interpreted as evidence that intestinal production represents a major source of circulating melatonin. The quantitative contribution of intestinal synthesis to systemic melatonin concentrations remains insufficiently established, particularly in humans [22,23,24].

Animal model studies have demonstrated a positive correlation between intestinal melatonin secretion and reduced inflammation in inflammatory bowel disease [21]. Egzogenous melatonin supplementation, in turn, had a beneficial effect on the gut microbiota profile, also contributing to the reduction in chronic inflammation [22,23]. Melatonin supplementation corrected the risk associated with an unhealthy diet in mice and partially restored the gut microbial composition and diversity toward that observed in healthy controls [19]. This experiment is another attempt to explain the mechanisms behind melatonin’s beneficial effects on body weight and metabolism.

Another important element of metabolic regulation by the biological clock is the assessment of the energy status of the cell using the so-called nutrient sensors, sirtuin 1 (SIRT1), AMPK and PGC1α [24,25,26,27,28]. SIRT1, a nicotinamide adenine dinucleotide (NAD+)-dependent deacetylase, is activated by nutrient deprivation and directly binds to CLOCK-BMAL1 and deacetylates PER2, causing its degradation [29]. SIRT1 plays an important metabolic role, and SIRT1 protein levels increase during periods of nutrient deprivation [30]. Among other things, SIRT1 reduces hepatic triglyceride levels by inhibiting lipogenesis and stimulating fatty acid β-oxidation [31]. It is also involved in insulin metabolism and regulating insulin secretion by pancreatic islet B cells [29,30,32,33]. Furthermore, SIRT1 regulates inflammatory processes and influences the maintenance of redox balance by influencing the expression of IL-6, TNF-α, and activation of superoxide dismutase (SOD) [32].

VAT represents one of the primary peripheral targets of circadian and metabolic regulation [34]. Compared with subcutaneous adipose tissue, VAT exhibits greater endocrine activity, higher immune cell infiltration, enhanced lipolytic activity, and increased secretion of pro-inflammatory adipokines [35,36]. Experimental evidence indicates that disruption of circadian rhythms and impaired melatonin signaling promote adipocyte hypertrophy, mitochondrial dysfunction, and chronic low-grade inflammation within VAT [37,38]. Consequently, preservation of circadian homeostasis may represent an important mechanism protecting visceral adipose tissue from metabolic dysfunction. Collectively, these observations support the concept that visceral adipose tissue functions as an integrative metabolic organ translating circadian, nutritional, and microbial signals into systemic cardiometabolic responses, thereby providing the biological basis for the proposed Gut–Melatonin–VAT Axis.

### 3.1. Melatonin as a Hormone Influencing Metabolism

Melatonin exerts many of its physiological effects through two high-affinity G protein-coupled receptors, MT1 and MT2, whose expression and functional effects vary across tissues. Both receptors are expressed in human white and brown adipose tissue, indicating that adipose tissue is a direct target of melatonin signaling [39]. Experimental studies have shown that melatonin can modulate adipocyte metabolism, including cAMP/cGMP signaling, GLUT4 expression and glucose uptake, as well as lipolysis [40,41]. More recently, experiments using human subcutaneous and visceral adipose tissue explants demonstrated that melatonin can modulate insulin signaling in a time-of-day-dependent manner, with reduced insulin sensitivity observed in visceral adipose tissue during the evening [42].

The clinical significance of MT2 melatonin receptor gene polymorphisms has also been confirmed. The SNP variant rs10830963 (C/G) was associated with observed elevated fasting glucose and a higher risk of developing type 2 diabetes [41,43]. The presence of the G allele in this gene results in impaired early-phase insulin secretion and reduced β-cell sensitivity to glucose [44], and also influences the rate of progression from prediabetes to diabetes [45].

Previous reports indicate a significant role of melatonin in the development and progression of metabolic syndrome features, including the ability of melatonin to modulate the expression of glucose transporters in adipose tissue [46,47], and studies on animal models have shown that melatonin supplementation limited the growth of visceral fat despite a high-calorie diet [48]. Also, another studies on animal models clearly confirm that one of the factors responsible for body weight gain is prolonged evening exposure to light resulting in reduced melatonin synthesis, and this effect can be reversed by appropriate supplementation [44,48,49].

Melatonin also interacts extensively with the previously mentioned SIRT1, SIRT3, and SIRT6 signaling pathway proteins. Its effect on Bax protein expression results in maintaining mitochondrial function, limiting the effects of oxidative stress, inhibiting apoptosis, and regulating autophagy. Furthermore, melatonin exhibits antioxidant properties not only by directly scavenging free radicals but also by activating the STAT3 antioxidant system in mitochondria [50]. Melatonin may exert a protective effect against obesity-induced cardiac remodeling through SIRT1-mediated activation of Nrf2 [51]. In cell line studies using a hydrogen peroxide-induced oxidative stress model, melatonin activated SIRT1 and SIRT3 while simultaneously inhibiting p66Shc, which resulted in reduced levels of reactive oxygen species and malondialdehyde within cells [52]. Malondialdehyde exhibits cytotoxic, mutagenic, and carcinogenic properties. They can also inhibit enzymes associated with defending cells against oxidative stress. Not only do these processes contribute to the development of many diseases, but they are also a part of the aging process [53]. The relationship between SIRT1 and melatonin is reciprocal, as this protein can directly influence melatonin secretion by the pineal gland. In summary, the complex interaction between melatonin and SIRT1 and SIRT3 appears to be a key modulator of various signaling pathways, with promising therapeutic implications for a wide range of diseases [54].

The importance of melatonin in obesity control is widely accepted, but the mechanisms underlying this relationship have not yet been fully described. Experimental studies in animal models have demonstrated, among other things, that chronic oral melatonin administration stimulates the browning of adipose tissue [55]. This phenomenon is metabolically beneficial because beige and brown adipose tissue are responsible for, among other things, increased energy expenditure in thermogenesis, improved glucose utilization, and decreased insulin resistance [43,56]. However, it remains unclear whether melatonin induces beige adipose tissue through de novo cell differentiation or through transdifferentiation of white adipocytes [57]. Another in vitro study demonstrated that melatonin has the ability to inhibit white adipogenesis [58]. Attempts were also made to determine the effect of melatonin on tissue insulin sensitivity. Zambrano’s study assessed the tissue response of visceral and subcutaneous adipose tissue cells to melatonin supplementation depending on the time of day, finding that melatonin reduced insulin sensitivity in the evening. These results may partially explain previous studies demonstrating a decrease in glucose tolerance after oral administration of melatonin in the evening or during a late meal, when endogenous melatonin is present in the body [46]. Clinical observations in a small (*n* = 46) group of obese women showed that melatonin supplementation at a dose of 6 mg per day for 40 days significantly improved glucose tolerance and adipokine secretion profile [59]. In another studies: melatonin supplementation reduces the expression of glucose transporter t.4 (GLUT-4) and glucose uptake by brown adipose tissue cells [41]. Furthermore, it prolongs the survival of beta-cells in pancreatic islets by inhibiting apoptosis in response to hyperglycemia [57].

### 3.2. Dietary Regulation of the Gut–Melatonin–VAT Axis

Dietary composition influences visceral adipose tissue (VAT) not only through energy balance but also by regulating endocrine and immune metabolic pathways that determine adipocyte function [60]. One potential mechanism linking nutrition with VAT homeostasis may involve interactions between intestinal melatonin signaling and adipose tissue endocrine function. Increasing evidence indicates that the gastrointestinal tract should be regarded as an important endocrine organ integrating nutritional signals with circadian regulation, immune responses, and adipose tissue metabolism [61].

Unlike pineal melatonin, whose secretion is primarily regulated by the light–dark cycle, gastrointestinal melatonin production is probably associated with food intake and nutrient-related signals. Enterochromaffin cells contribute to gastrointestinal melatonin synthesis, which is linked to the tryptophan–serotonin pathway [62]. Thus, nutrient availability may influence gastrointestinal melatonin synthesis, although the magnitude and physiological relevance of this effect appear to depend on the nutrient and experimental model. Tryptophan-containing foods provide the amino acid precursor for serotonin and melatonin biosynthesis; however, evidence that habitual dietary tryptophan intake directly increases intestinal melatonin production remains limited. Moreover, several micronutrients participate in pathways related to serotonin and melatonin biosynthesis, with vitamin B6 playing a particularly important role in serotonin synthesis.

Beyond individual nutrients, dietary patterns may influence several components of the gut–melatonin–VAT axis, including gut microbiota composition, inflammatory signaling, and circadian metabolic regulation. The Mediterranean diet, characterized by high consumption of vegetables, fruits, legumes, olive oil, whole grains, and polyphenol-rich foods, has consistently been associated with reduced systemic inflammation, improved gut microbiota composition, and enhanced circadian metabolic regulation [63]. Polyphenols, including resveratrol and quercetin, can activate antioxidant and metabolic pathways in adipose tissue, including AMPK-, SIRT1-, and Nrf2-related signaling, which may complement the antioxidant and mitochondrial effects of melatonin [64,65]. Conversely, Western dietary patterns rich in saturated fat, refined carbohydrates, and ultra-processed foods promote gut dysbiosis, increased intestinal permeability, and chronic low-grade inflammation, conditions that may impair intestinal melatonin synthesis and disrupt its physiological protective functions [64,66].

Melatonin influences VAT through several complementary mechanisms: adipocyte differentiation, mitochondrial metabolism, insulin sensitivity, inflammatory responses [67,68]. These effects are mediated through activation of MT1 and MT2 receptors expressed in adipose tissue as well as through receptor-independent free radical scavenging and modulation of intracellular signaling pathways involving AMP-activated protein kinase (AMPK), sirtuin-1 (SIRT1), nuclear factor erythroid 2-related factor 2 (Nrf2), and nuclear factor-κB (NF-κB) [27,69]. Collectively, these mechanisms reduce oxidative stress, suppress inflammatory cytokine production, and improve adipocyte metabolic flexibility [70].

An important but often underappreciated aspect of melatonin action involves regulation of adipokine secretion [71]. Visceral adipose tissue functions as an active endocrine organ producing numerous bioactive mediators that influence whole-body metabolism: increased production of leptin, resistin, and visfatin, together with pro-inflammatory cytokines such as TNF-α and IL-6, and reduced production of the insulin-sensitizing adipokine adiponectin (Table 1). This shift toward a pro-inflammatory adipokine profile contributes to insulin resistance, endothelial dysfunction, hepatic steatosis, and cardiovascular complications.

Experimental studies suggest that melatonin partially reverses this adverse secretory phenotype [71]. Melatonin supplementation has been shown to reduce leptin, TNF-α, IL-6, and MCP-1, while other experimental studies indicate restoration of adiponectin expression in obese adipose tissue [72]. Adiponectin is particularly relevant because it promotes AMPK activation, fatty acid oxidation and insulin sensitivity while exerting anti-inflammatory effects. Melatonin has also been reported to attenuate macrophage infiltration into VAT and promote polarization toward anti-inflammatory M2 macrophages, thereby further reducing adipose tissue inflammation [73].

**Table 1:** Adipokines regulated by melatonin and their metabolic significance. “↑” is increasing by melatonin; “↓” is decreasing.

Adipokine	Effect of Melatonin	Clinical Relevance	References
Adiponectin	↑ expression/secretion/level	↑ insulin sensitivity; AMPK activation; anti-inflammatory effects	[48,74]
Leptin	↓ level/signaling	Regulation of energy homeostasis; leptin resistance in obesity	[71,73,74]
Resistin	↓ level	Associated with insulin resistance and inflammation	[74]
Omentin	↑ level	Insulin-sensitizing and anti-inflammatory effects	[75]
Visfatin	↓	Pro-inflammatory and metabolic effects	[74]
TNF-α	↓	Adipose inflammation; insulin resistance	[71,73]
IL-6	↓	Chronic inflammation; metabolic dysfunction	[71]
MCP-1/CCL2	↓	Monocyte recruitment and macrophage infiltration	[71]

In summary, melatonin may partially reverse the obesity-associated adipokine and inflammatory phenotype of visceral adipose tissue, including attenuation of leptin, TNF-α, IL-6, and MCP-1 signaling and, in some experimental models, restoration of adiponectin expression. These effects are associated with reduced adipose tissue inflammation and improved metabolic homeostasis, although the effects of melatonin on adipose insulin sensitivity may depend on circadian timing and metabolic context [46].

Emerging evidence suggests that many of these endocrine effects may be indirectly mediated by the gut microbiota [12,16]. Dietary fiber fermentation generates short-chain fatty acids (SCFAs), particularly butyrate, acetate, and propionate, which enhance intestinal barrier integrity, stimulate glucagon-like peptide-1 (GLP-1) secretion, and may reduce metabolic endotoxemia [6,7,64,70,74]. SCFAs may also influence enterochromaffin cell activity and intestinal melatonin synthesis, thereby establishing a bidirectional interaction between dietary nutrients, gut microbiota, and melatonin production [76,77]. Conversely, impaired melatonin signaling associated with circadian disruption or an unhealthy diet may exacerbate gut dysbiosis and intestinal inflammation, contributing to altered adipokine secretion and VAT dysfunction [11,13,15,19,20,71].

Taken together, current evidence supports the concept that dietary regulation of intestinal melatonin may represents an important mechanistic link connecting nutrition with visceral adipose tissue biology [16,19,20,71]. Rather than acting solely as an antioxidant hormone, intestinal melatonin appears to function as an integrative metabolic signal coordinating gut microbial homeostasis, adipokine secretion, inflammatory activity, and adipocyte metabolism [16,20,71,73,74]. Future clinical studies should determine whether dietary interventions designed to enhance endogenous intestinal melatonin production can improve adipokine profiles and reduce visceral adiposity, potentially offering a novel strategy for the prevention of obesity-related cardiometabolic disorders.

## 4. Mechanisms Linking Gut Microbiota and VAT

Obesity is frequently associated with reduced microbial diversity and altered taxonomic composition [78]. Although early studies focused on changes in the Firmicutes-to-Bacteroidetes ratio, contemporary research indicates that microbial functionality may be more important than simple taxonomic shifts [79].

Dysbiosis is characterized by reduced abundance of beneficial SCFA-producing bacteria and increased prevalence of pro-inflammatory microbial communities [70,80]. These changes contribute to impaired gut barrier function, chronic low-grade inflammation, altered energy metabolism, and enhanced fat storage [6,81].

Recent metagenomic studies suggest that gut microbiota composition is associated with visceral fat accumulation, highlighting the potential value of microbiome-derived markers for metabolic risk assessment [82].

### 4.1. Intestinal Barrier Dysfunction and Metabolic Endotoxemia

One of the best-characterized pathways connecting gut microbiota and VAT involves disruption of intestinal barrier integrity [6,81]. Under physiological conditions, the intestinal epithelium prevents excessive translocation of microbial products into systemic circulation. However, dietary factors, obesity, and dysbiosis may impair tight-junction function and increase intestinal permeability [81,83]. As a consequence, bacterial lipopolysaccharide (LPS) can enter the circulation, leading to a phenomenon known as metabolic endotoxemia. Metabolic endotoxemia activates TLR4/NF-κB signaling, promoting macrophage recruitment, adipose tissue inflammation, and impaired insulin signaling within VAT [84]. Within VAT, these inflammatory processes promote macrophage recruitment, adipocyte dysfunction, and impaired insulin signaling, thereby contributing to metabolic syndrome development.

### 4.2. Immune Regulation and VAT Inflammation

In obesity, adipose tissue undergoes profound immune remodeling characterized by infiltration of pro-inflammatory macrophages and T lymphocytes [39,40].

The gut microbiota influences systemic immune responses through microbial metabolites, bacterial antigens, and interactions with intestinal immune cells. Dysbiosis promotes a pro-inflammatory environment favoring M1 macrophage polarization and cytokine production, whereas beneficial microbial communities stimulate regulatory T cells and anti-inflammatory M2 macrophages [6]. This immune modulation represents a critical mechanism through which gut microbiota contributes to VAT inflammation and metabolic dysfunction.

### 4.3. Beneficial Bacteria and Short-Chain Fatty Acids

Not all microbial taxa exert detrimental effects. Several bacterial genera, including *Lactobacillus*, *Bifidobacterium*, *Faecalibacterium*, and *Akkermansia*, have been associated with favorable metabolic outcomes and reduced visceral adiposity.

*Faecalibacterium prausnitzii*

*Faecalibacterium prausnitzii* is one of the most abundant butyrate-producing bacteria in the healthy human intestine [81]. Reduced abundance of this species has been observed in obesity, insulin resistance, and inflammatory disorders [85]. Through butyrate production, *F. prausnitzii* supports intestinal barrier function and suppresses inflammatory signaling pathways [86,87].

*Akkermansia muciniphila*

*Akkermansia muciniphila* plays a key role in maintaining the intestinal mucus layer. Numerous studies have reported inverse associations between its abundance and obesity-related parameters, including VAT accumulation [88]. Clinical trials suggest that supplementation with *A. muciniphila* may improve selected metabolic parameters, although effects on whole-body insulin sensitivity appear to be context-dependent [89,90].

**Short-Chain Fatty Acids (SCFAs)**

SCFAs, particularly acetate, propionate, and butyrate, represent major mediators of microbiota-host interactions [Table 2]. These metabolites activate G-protein-coupled receptors (GPR41 and GPR43) and stimulate the secretion of glucagon-like peptide-1 (GLP-1) and peptide YY (PYY), thereby contributing to appetite regulation and glucose homeostasis [91,92]. Moreover, butyrate enhances intestinal barrier integrity by supporting tight-junction proteins and reducing systemic exposure to LPSs [93,94]. Consequently, adequate SCFA production may contribute to protection against VAT accumulation and metabolic dysfunction.

Collectively, these findings indicate that microbial metabolites, intestinal barrier integrity, and immune regulation constitute major mechanisms through which the gut microbiota influences visceral adipose tissue. Importantly, many of these processes are themselves regulated by dietary timing and circadian rhythms, providing the rationale for chrononutrition-based interventions [101].

## 5. Chrononutrition

The concept of chrononutrition stems from observations indicating a significant impact of meal times on metabolic health [102]. Observational and experimental studies indicate that the timing of energy intake may influence body weight and metabolic outcomes, with later eating patterns generally associated with less favorable metabolic profiles [103]. An evening chronotype has been associated with a higher prevalence of overweight and obesity and poorer metabolic health, despite comparable total energy intake in some populations [104]. According to the historical definition, chronotype is a biological characteristic leading interindividual differences in the circadian phase relative to the light–dark cycle. Chronotypes are commonly categorized as morning (MT), evening (ET), or intermediate (IT) types. Morning types prefer activities at the beginning of the day, and evening types prefer main activities in the late afternoon or evening [105].

Key cellular sensors linking nutritional status to circadian regulation include the AMP/ATP and NAD+/NADH ratios [106]. An increase in the AMP/ATP ratio during energy stress activates AMPK, whereas changes in the NAD+/NADH redox state influence sirtuin activity; both pathways interact with the molecular circadian clock [107,108]. Inappropriate feeding patterns can disrupt peripheral circadian clocks, whereas appropriately timed food intake can entrain and synchronize peripheral clocks [105].

In response to these reports, several dietary concepts have emerged that consider the role of meal timing throughout the day [106]. Chrononutrition approaches include time-restricted eating, early time-restricted eating, intermittent fasting, and other dietary patterns that align food intake with the circadian cycle [109]. Intermittent fasting has been associated with improvements in several metabolic and inflammatory markers, while emerging evidence suggests that it may also modulate the gut microbiota; however, evidence for microbiota-mediated effects in humans remains limited [110].

## 6. Therapeutic Perspectives

Growing understanding of the Gut–Melatonin–VAT Axis has stimulated interest in interventions targeting gut microbial composition, intestinal barrier integrity, microbial metabolite production, and circadian regulation. Dietary fiber, prebiotics, probiotics, synbiotics, and postbiotics may modulate gut microbial composition and metabolite production, potentially supporting intestinal barrier integrity and reducing low-grade inflammation [111].

Personalized nutrition and fecal microbiota transplantation are also being investigated, although their role in the prevention and treatment of obesity-related metabolic disorders remains to be established. Among potential microbial targets, *Akkermansia muciniphila* and *Faecalibacterium prausnitzii* are of particular interest because of their associations with intestinal barrier integrity, reduced low-grade inflammation, and improved metabolic profiles [112].

Experimental studies suggest that melatonin supplementation may improve metabolic homeostasis through effects on circadian regulation, intestinal barrier function, gut microbiota composition, and adipose tissue inflammation [13,19,20].

Furthermore, chrononutritional interventions may influence gastrointestinal microbial composition and rhythmicity, potentially contributing to metabolic benefits. Circadian misalignment has been associated with alterations in gut microbial composition and diversity [106].

Intermittent fasting and caloric restriction induce beneficial metabolic adaptations through cellular pathways involving mTOR, sirtuins, AMPK, and redox homeostasis, including the stimulation of autophagy, the preservation of mitochondrial function, the removal of damaged intracellular components, and improved glucose and lipid metabolism [113]. The beneficial metabolic effect and influence on body weight control apply to both individuals with a baseline normal body weight and obese patients [114,115].

Western dietary patterns characterized by high consumption of energy-dense, fat- and sugar-rich foods may impair intestinal barrier integrity and promote gut dysbiosis, contributing to adipose tissue inflammation through microbiota-dependent mechanisms [97]. In addition, late-day energy intake and circadian misalignment may adversely affect metabolic regulation and gut microbial rhythmicity [106,108]. These alterations may contribute to hepatic and systemic metabolic dysfunction through increased intestinal permeability and microbiota-derived signals [116].

Summary od these perspectives are in Table 3.

## 7. Conclusions

The present review highlights the complex interactions between nutrition, circadian regulation, intestinal melatonin secretion, gut microbiota, and visceral adipose tissue in maintaining metabolic homeostasis. Rather than acting as independent regulators, these factors constitute an interconnected biological network that may influence obesity development and its cardiometabolic complications.

Based on the available evidence, we propose the Gut–Melatonin–VAT Axis as a conceptual framework integrating dietary composition, meal timing, intestinal melatonin, microbial homeostasis, and visceral adipose tissue function. This model provides a broader perspective on the mechanisms linking lifestyle factors with adipose tissue biology and systemic metabolism.

Although many mechanistic observations originate from experimental studies, accumulating clinical evidence supports the relevance of this integrated pathway in humans. Future research should aim to identify reliable biomarkers of Gut–Melatonin–VAT Axis dysfunction and determine whether personalized nutritional, chrononutritional, microbiota-targeted, or melatonin-based interventions may improve metabolic health and reduce obesity-related cardiometabolic risk.

## Figures and Tables

**Table 2:** The principal short-chain fatty acids produced by the gut microbiota and their effects on visceral adipose tissue and metabolic regulation.

SCFA	Key Molecular Mechanisms	Metabolic Effects (VAT and Systemic)	Ref.
**Acetate**	Activation of **GPR43/FFAR2**; inhibition of excessive adipocyte lipolysis; modulation of insulin-dependent lipid handling	Regulation of adipose lipid metabolism; suppression of excessive lipolysis and circulating FFA availability; modulation of insulin-mediated fat accumulation	[95,96,97]
**Propionate**	Activation of **GPR41/FFAR3** and **GPR43/FFAR2**; stimulation of intestinal gluconeogenesis via a gut–brain–portal neural circuit	Increased GLP-1 and PYY secretion; enhanced satiety; improved glucose homeostasis and insulin sensitivity	[95,97,98]
**Butyrate**	Stabilization of **HIF-1α**; enhancement of tight-junction proteins; HDAC inhibition; support of epithelial energy metabolism	Maintenance of intestinal barrier integrity; reduced permeability and bacterial/LPS translocation; attenuation of intestinal and systemic inflammation	[95,99,100]

**Table 3:** Potential therapeutic strategies targeting the Gut–Melatonin–VAT Axis. “↑” is increasing; “↓” is decreasing.

Intervention	Microbiota	Melatonin/Circadian Regulation	VAT/Metabolic Effects	Evidence	Ref.
Mediterranean diet	↑ microbial diversity and beneficial taxa; ↑ SCFA production	Potential support of circadian metabolic regulation	↓ inflammation; improved metabolic profile; potential reduction in visceral adiposity	Strong	[63,65]
High-fiber diet	↑ SCFA-producing bacteria and SCFA availability	Indirect effects through microbial–circadian signaling	Improved metabolic health; potential reduction in VAT accumulation	Strong	[74,76,108]
Tryptophan	Modulation of tryptophan metabolism by microbiota	↑ substrate availability for melatonin synthesis	Potentially ↑ adiponectin/improved metabolic signaling	Emerging	[61,62]
Polyphenols	↑ beneficial taxa, including *Akkermansia*	↑ antioxidant/metabolic signaling; potentially supports melatonin pathways	↓ oxidative stress and inflammation	Emerging	[64,65]
Time-restricted eating	May restore microbial rhythmicity	↑ circadian alignment; effects depend on timing	↓ body weight and improved insulin sensitivity in some studies	Moderate	[106]
Intermittent fasting	May modulate microbial composition	May improve circadian/metabolic signaling	↓ body weight; improved glucose and lipid metabolism	Moderate	[107,110,111,112]
Melatonin supplementation	May modulate microbial composition and rhythmicity	Direct restoration of melatonin signaling	Potential ↓ VAT accumulation, oxidative stress and inflammation	Moderate/preclinical	[11,14,16,67,69]
Probiotics	Modulation of microbial composition/function	Indirect	Potential improvement in metabolic parameters and inflammation	Moderate	[95,108]
Synbiotics	↑ beneficial bacteria and microbial metabolites	Indirect	Potential metabolic and anti-inflammatory effects	Emerging	[95]
Postbiotics	Provide microbial-derived metabolites/components	Indirect	Potential effects on barrier function, inflammation and metabolic health	Emerging	[113]

## Data Availability

No new data were created or analyzed in this study. Data sharing is not applicable to this article.

## References

[1] Peri K., Eisenberg M. (2024). Review on the update in obesity management: Epidemiology. BMJ Public Health.

[2] Lin J.C., Tandar C.E. (2026). Global patterns of visceral adiposity and cardiometabolic disease. Nat. Rev. Endocrinol..

[3] Sam S. (2018). Differentia effect of subcutaneous abdominal and visceral adipose tissue on cardiometabolic risk. Horm. Mol. Biol. Clin. Investig..

[4] Lee M.-J., Kim J. (2024). The pathophysiology of visceral adipose tissues in cardiometabolic diseases. Biochem. Pharmacol..

[5] Shetty S., Suvarna R., Bhattacharya S., Seetharaman K. (2025). Visceral Adiposity and Cardiometabolic Risk: Clinical Insights and Assessment. Cardiol. Rev..

[6] Rosendo-Silva D., Viana S., Carvalho E. (2023). Are gut dysbiosis, barrier disruption, and endotoxemia related to adipose tissue dysfunction in metabolic disorders? Overview of the mechanisms involved. Intern Emerg. Med..

[7] Litichevskiy L., Thaiss C.A. (2022). The Oscillating Gut Microbiome and Its Effects on Host Circadian Biology. Annu. Rev. Nutr..

[8] Makki K., Froguel P., Wolowczuk I. (2013). Adipose Tissue in Obesity-Related Inflammation and Insulin Resistance: Cells, Cytokines, and Chemokines. Mediat. Inflamm..

[9] Thaiss C.A., Zeevi D., Levy M. (2014). Transkingdom control of microbiota diurnal oscillations promotes metabolic homeostasis. Cell.

[10] Zarrinpar A., Chaix A., Yooseph S., Panda S. (2014). Diet and Feeding Pattern Affect the Diurnal Dynamics of the Gut Microbiome. Cell Metab..

[11] Voigt R.M., Forsyth C.B., Green S.J. (2014). Circadian disorganization alters intestinal microbiota. PLoS ONE.

[12] Lotti S., Dinu M., Colombini B., Amedei A., Sofi F. (2023). Circadian rhythms, gut microbiota, and diet: Possible implications for health. Nutr. Metab. Cardiovasc. Dis..

[13] Yin J., Li Y., Han H., Ma J., Liu G., Wu X., Huang X., Fang R., Baba K., Bin P. (2020). Administration of Exogenous Melatonin Improves the Diurnal Rhythms of the Gut Microbiota in Mice Fed a High-Fat Diet. mSystems.

[14] Li W., Wang Z., Cao J., Dong Y., Chen Y. (2023). Melatonin improves the homeostasis of mice gut microbiota rhythm caused by sleep restriction. Microbes Infect..

[15] Vohra A., Karnik R., Desai M., Vyas H., Kulshrestha S., Upadhyay K.K., Devkar R. (2024). Melatonin-mediated corrective changes in gut microbiota of experimentally chronodisrupted C57BL/6J mice. Chronobiol. Int..

[16] Bonmatí-Carrión M.A., Rol M.A. (2024). Melatonin as a Mediator of the Gut Microbiota–Host Interaction: Implications for Health and Disease. Antioxidants.

[17] Zheng J., Zhou Y., Zhang D., Ma K., Gong Y., Luo X., Liu J., Cui S. (2024). Intestinal melatonin levels and gut microbiota homeostasis are independent of the pineal gland in pigs. Front. Microbiol..

[18] Yin J., Li Y., Han H., Chen S., Gao J., Liu G., Wu X., Deng J., Yu Q., Huang X. (2018). Melatonin reprogramming of gut microbiota improves lipid dysmetabolism in high-fat diet-fed mice. J. Pineal Res..

[19] Xu P., Wang J., Hong F. (2017). Melatonin prevents obesity through modulation of gut microbiota in mice. J. Pineal Res..

[20] Rong B., Wu Q., Reiter R.J., Sun C. (2021). The Mechanism of Oral Melatonin Ameliorates Intestinal and Adipose Lipid Dysmetabolism Through Reducing Escherichia Coli-Derived Lipopolysaccharide. Cell Mol. Gastroenterol. Hepatol..

[21] Zhao Z.-X., Yuan X., Cui Y.-Y., Liu J., Shen J., Jin B.-Y., Feng B.-C., Zhai Y.-J., Zheng M.-Q., Kou G.-J. (2021). Melatonin Mitigates Oxazolone-Induced Colitis in Microbiota-Dependent Manner. Front. Immunol..

[22] Monk J.M., Lepp D., Zhang C.P., Wu W., Zarepoor L., Lu J.T., Pauls K.P., Tsao R., Wood G.A., Robinson L.E. (2016). Diets Enriched with Cranberry Beans Alter the Microbiota and Mitigate Colitis Severity and Associated Inflammation. J. Nutr. Biochem..

[23] Iesanu M.I., Zahiu C.D.M., Dogaru I.-A., Chitimus D.M., Pircalabioru G.G., Voiculescu S.E., Isac S., Galos F., Pavel B., O’Mahony S.M. (2022). Melatonin–Microbiome Two-Sided Interaction in Dysbiosis-Associated Conditions. Antioxidants.

[24] Asher G., Gatfield D., Stratmann M. (2008). SIRT1 regulates circadian clock gene expression through PER2 deacetylation. Cell.

[25] Liang F., Kume S., Koya D. (2009). SIRT1 and insulin resistance. Nat. Rev. Endocrinol..

[26] Kemper J.K., Choi S.E., Kim D.H. (2013). Sirtuin 1 Deacetylase.

[27] Acosta-Rodríguez V.A., Rijo-Ferreira F., Green C.B., Takahashi J.S. (2021). Importance of circadian timing for aging and longevity. Nat. Commun..

[28] Yang Y., Liu Y., Wang Y., Chao Y., Zhang J., Jia Y. (2022). Regulation of SIRT1 and Its Roles in Inflammation. Front. Immunol..

[29] Nakahata Y., Sahar S., Astarita G. (2009). Circadian control of the NAD+ salvage pathway by CLOCK-SIRT1. Science.

[30] Hayashida S., Arimoto A., Kuramoto Y. (2010). Fasting promotes the expression of SIRT1, an NAD+-dependent protein deacetylase, via activation of PPARalpha in mice. Mol. Med. Rep..

[31] Purushotham A., Schug T.T., Xu Q. (2009). Hepatocyte-specific deletion of SIRT1 alters fatty acid metabolism and results in hepatic steatosis and inflammation. Cell Metab..

[32] Bordone L., Motta M.C., Picard F. (2006). Sirt1 regulates insulin secretion by repressing UCP2 in pancreatic β cells. PLoS Biol..

[33] Luu L., Dai F.F., Prentice K.J. (2013). The loss of Sirt1 in mouse pancreatic beta cells impairs insulin secretion by disrupting glucose sensing. Diabetologia.

[34] Ando H., Yanagihara H., Hayashi Y. (2005). Rhythmic messenger ribonucleic acid expression of clock genes and adipocytokines in mouse visceral adipose tissue. Endocrinology.

[35] Khan S.K., Chan Y.T., Revelo X.S., Winer D.A. (2020). The Immune Landscape of Visceral Adipose Tissue During Obesity and Aging. Front. Endocrinol..

[36] Guzik T.J., Skiba D.S., Touyz R.M., Harrison D.G. (2017). The role of infiltrating immune cells in dysfunctional adipose tissue. Cardiovasc. Res..

[37] Xiong X., Lin Y., Lee J., Paul A., Yechoor V., Figueiro M., Ma K. (2021). Chronic circadian shift leads to adipose tissue inflammation and fibrosis. Mol. Cell Endocrinol..

[38] Walkiewicz K.W., Dzięgielewska-Gęsiak S., Myrcik D., Bednarczyk M., Muc-Wierzgoń M. (2025). Eating behavior patterns in relation to obesity phenotypes and beige adipose tissue content with a focus on young women; a narrative review. Front. Nutr..

[39] Brydon L., Petit L., Delagrange P., Strosberg A.D., Jockers R. (2001). Functional expression of MT2 (Mel1b) melatonin receptors in human PAZ6 adipocytes. Endocrinology.

[40] Zalatan F., Krause J.A., Blask D.E. (2001). Inhibition of isoproterenol-induced lipolysis in rat inguinal adipocytes in vitro by physiological melatonin via a receptor-mediated mechanism. Endocrinology.

[41] Prokopenko I., Langenberg C., Florez J. (2009). Variants in MTNR1B influence fasting glucose levels. Nat. Genet..

[42] Zambrano C., Tena Garitaonaindia M., Salmerón D. (2024). Melatonin decreases human adipose tissue insulin sensitivity. J. Pineal Res..

[43] Lyssenko V., Nagorny C., Erdos M. (2009). Common variant in MTNR1B associated with increased risk of type 2 diabetes and impaired early insulin secretion. Nat. Genet..

[44] Tan D.--X., Manchester L.C., Fuentes-Broto L., Paredes S.D., Reiter R.J. (2011). Significance and application of melatonin in the regulation of brown adipose tissue metabolism: Relation to human obesity. Obes. Rev..

[45] Walford G.A., Green T., Neale B., Isakova T., Rotter J.I., Grant S.F.A., Fox C.S., Pankow J.S., Wilson J.G., Meigs J.B. (2012). Common genetic variants differentially influence the transition from clinically defined states of fasting glucose metabolism. Diabetologia.

[46] Nduhirabandi F., du Toit E.F., Lochner A. (2012). Melatonin and the metabolic syndrome: A tool for effective therapy in obesity-associated abnormalities?. Acta Physiol..

[47] Heo J., Yoon D.W., Yu J.H., Kim N.H., Yoo H.J., Seo J.A., Kim S.G., Choi K.M., Baik S.H., Choi D.S. (2018). Melatonin improves insulin resistance and hepatic steatosis through attenuation of alpha-2-HS-glycoprotein. J. Pineal Res..

[48] Korhonen T., Mustonen A.M., Nieminen P., Saarela S. (2008). Effects of cold exposure, exogenous melatonin and short-day treatment on the weight-regulation and body temperature of the Siberian hamster (*Phodopus sungorus*). Regul. Pept..

[49] Wade G.N., Bartness T.J. (1984). Seasonal obesity in Syrian hamsters: Effects of age, diet, photoperiod, and melatonin. Am. J. Physiol..

[50] Yang Y., Duan W., Jin Z., Yi W., Yan J., Zhang S., Wang N., Liang Z., Li Y., Chen W. (2013). JAK2/STAT3 activation by melatonin attenuates the mitochondrial oxidative damage induced by myocardial ischemia/reperfusion injury. J. Pineal Res..

[51] Zhang W., Wang X., Tang Y., Huang C. (2023). Melatonin alleviates doxorubicin-induced cardiotoxicity via inhibiting oxidative stress, pyroptosis and apoptosis by activating Sirt1/Nrf2 pathway. BioMed Pharmacother..

[52] Liu L., Xia G., Li P., Wang Y., Zhao Q. (2021). Sirt-1 Regulates Physiological Process and Exerts Protective Effects against Oxidative Stress. BioMed. Res. Int..

[53] Całyniuk B., Grochowska-Niedworok E., Walkiewicz K., Kawecka S., Popiołek E., Fatyga E. (2016). Malondialdehyde (MDA)- product of lipid peroxidation as marker of homeostasis disorders and aging. Ann. Acad. Medicae Silesiensis.

[54] Hosseinzadeh A., Seinafianpour F., Sheibani M., Taheri M., Naeini A., Reiter R., Mehrzadi S. (2025). Therapeutic Aspects of Melatonin-sirtuin Crosstalk: An Updated Review of Current Data Based on Cellular Mechanisms. Curr. Drug Targets.

[55] Salagre D., Chayah M., Molina-Carballo A., Oliveras-Lopez M.-J., Munos-Hoyos A., Navarro-Alarcon M., Fernandez-Vasqez G., Agil A. (2022). Melatonin induces fat browning by transdifferentiation of white adipocytes and de novo differentiation of mesenchymal stem cells. Food Funct..

[56] Peirce V., Pellegrinelli V., Vidal-Puig A., AhimaR (2015). Adipose structure (white, brown, beige). Metabolic Syndrome.

[57] Costes S., Boss M., Thomas A.P., Matveyenko A.V. (2015). Activation of Melatonin Signaling Promotes β-Cell Survival and Function. Mol. Endocrinol..

[58] Alonso-Vale M.I., Peres S.B., Vernochet C., Farmer S.R., Lima F.B. (2009). Adipocyte differentiation is inhibited by melatonin through the regulation of C/EBPbeta transcriptional activity. J. Pineal Res..

[59] Alamdari M.N., Shabestari A.N., Najafipour F., Mirmazhari (2024). Melatonin supplementation improves glycemic hemostasis, lipid profile, and adipokine concentrations of obese women: A double-blind randomized clinical trial. Biomed. Adv..

[60] Tran H.Q., Bretin A., Adeshirlarijaney A., Yeoh B.S., Vijay-Kumar M., Zou J., Denning T.L., Chassaing B., Gewirtz A.T. (2020). Western Diet”-Induced Adipose Inflammation Requires a Complex Gut Microbiota. Cell Mol. Gastroenterol. Hepatol..

[61] Zhao F., Ma C., Zhao G., Wang G., Li X., Yang K. (2019). Rumen-Protected 5-Hydroxytryptophan Improves Sheep Melatonin Synthesis in the Pineal Gland and Intestinal Tract. Med. Sci. Monit..

[62] Herrero M.J., Martínez F.J., Míguez J.M., Madrid J.A. (2007). Response of plasma and gastrointestinal melatonin to dietary supplementation with tryptophan and melatonin. J. Comp. Physiol. B.

[63] Haskey N., Estaki M., Ye J., Shim R.K., Singh S., Dieleman L.A., Jacobson K., Gibson D.L. (2023). A Mediterranean Diet Pattern Improves Intestinal Inflammation Concomitant with Reshaping of the Bacteriome in Ulcerative Colitis: A Randomised Controlled Trial. J. Crohns Colitis.

[64] Guan Q., Wang Z., Cao J., Dong Y., Chen Y. (2021). Mechanisms of Melatonin in Obesity: A Review. Int. J. Mol. Sci..

[65] Flori L., Piragine E., Spezzini J., Citi V., Calderone V., Martelli A. (2023). Influence of Polyphenols on Adipose Tissue: Sirtuins as Pivotal Players in the Browning Process. Int. J. Mol. Sci..

[66] Ding S., Chi M.M., Scull B.P., Rigby R., Schwerbrock N.M., Magness S., Jobin C., Lund P.K. (2010). High-fat diet: Bacteria interactions promote intestinal inflammation which precedes and correlates with obesity and insulin resistance in mouse. PLoS ONE.

[67] Farias T.D.S.M., Paixao R.I.D., Cruz M.M., de Sa R.D.C.D.C., Simão J.J., Antraco V.J., Alonso-Vale M.I.C. (2019). Melatonin Supplementation Attenuates the Pro-Inflammatory Adipokines Expression in Visceral Fat from Obese Mice Induced by A High-Fat Diet. Cells.

[68] Gao X.Y., Deng B.H., Li X.R., Wang Y., Zhang J.X., Hao X.Y., Zhao J.X. (2021). Melatonin Regulates Differentiation of Sheep Brown Adipocyte Precursor Cells *Via* AMP-Activated Protein Kinase. Front Vet. Sci..

[69] Liu Z., Gan L., Zhang T., Ren Q., Sun C. (2018). Melatonin alleviates adipose inflammation through elevating α-ketoglutarate and diverting adipose-derived exosomes to macrophages in mice. J. Pineal Res..

[70] Xu L., Li D., Li H. (2022). Suppression of obesity by melatonin through increasing energy expenditure and accelerating lipolysis in mice fed a high-fat diet. Nutr. Diabetes.

[71] Favero G., Stacchiotti A., Castrezzati S., Bonomini F., Albanese M., Rezzani R., Rodella L.F. (2015). Melatonin reduces obesity and restores adipokine patterns and metabolism in obese (ob/ob) mice. Nutr. Res..

[72] Ji S.M., Yoo H., Kim J.I., Choi M.J., Cheon H.G. (2025). Melatonin induces white-to-beige adipocyte transdifferentiation through melatonin receptor 1-mediated direct browning and indirect M2 polarization. Mol. Cell. Endocrinol..

[73] Song Y.M., Chen M.D. (2009). Effects of melatonin administration on plasma leptin concentration and adipose tissue leptin secretion in mice. Acta Biol. Hung..

[74] Bodnaruc A.M., Prud’homme D., Blanchet R., Giroux I. (2016). Nutritional modulation of endogenous glucagon-like peptide-1 secretion: A review. Nutr. Metab..

[75] Szewczyk-Golec K., Rajewski P., Gackowski M., Mila-Kierzenkowska C., Wesołowski R., Sutkowy P., Pawłowska M., Woźniak A. (2017). Melatonin Supplementation Lowers Oxidative Stress and Regulates Adipokines in Obese Patients on a Calorie-Restricted Diet. Oxid. Med. Cell Longev..

[76] Wang B., Zhu S., Liu Z., Wei H., Zhang L., He M., Pei F., Zhang J., Sun Q., Duan L. (2020). Increased Expression of Colonic Mucosal Melatonin in Patients with Irritable Bowel Syndrome Correlated with Gut Dysbiosis. Genom. Proteom. Bioinform..

[77] Song L., He M., Sun Q., Wang Y., Zhang J., Fang Y., Liu S., Duan L. (2021). *Roseburia hominis* Increases Intestinal Melatonin Level by Activating p-CREB-AANAT Pathway. Nutrients.

[78] U-Din M., Ahmed B.A., Syed S.A., Ong F.J., Oreskovich S.M., Gunn E., Surette M.G., Punthakee Z., Steinberg G.R., Morrison K.M. (2024). Characteristics of Abdominal Visceral Adipose Tissue, Metabolic Health and the Gut Microbiome in Adults. J. Clin. Endocrinol. Metab..

[79] Sun J., Germain A., Kaglan G., Servant F., Lelouvier B., Federici M., Fernandez-Real J.M., Sala D.T., Neagoe R.M., Bouloumié A. (2023). The visceral adipose tissue bacterial microbiota provides a signature of obesity based on inferred metagenomic functions. Int. J. Obes..

[80] Kim K.A., Gu W., Lee I.A., Joh E.H., Kim D.H. (2012). High fat diet-induced gut microbiota exacerbates inflammation and obesity in mice via the TLR4 signaling pathway. PLoS ONE.

[81] Miquel S., Martín R., Rossi O., Bermúdez-Humarán L.G., Chatel J.M., Sokol H., Thomas M., Wells J.M., Langella P. (2013). Faecalibacterium prausnitzii and human intestinal health. Curr. Opin. Microbiol..

[82] Nie X., Chen J., Ma X., Ni Y., Shen Y., Yu H., Panagiotou G., Bao Y. (2020). A metagenome-wide association study of gut microbiome and visceral fat accumulation. Comput. Struct. Biotechnol. J..

[83] Yan H., Qin Q., Chen J., Yan S., Li T., Gao X., Yang Y., Li A., Ding S. (2022). Gut Microbiome Alterations in Patients With Visceral Obesity Based on Quantitative Computed Tomography. Front. Cell Infect. Microbiol..

[84] Beisner J., Filipe Rosa L., Kaden-Volynets V., Stolzer I., Günther C., Bischoff S.C. (2021). Prebiotic Inulin and Sodium Butyrate Attenuate Obesity-Induced Intestinal Barrier Dysfunction by Induction of Antimicrobial Peptides. Front. Immunol..

[85] Munukka E., Rintala A., Toivonen R., Nylund M., Yang B., Takanen A., Hänninen A., Vuopio J., Huovinen P., Jalkanen S. (2017). Faecalibacterium prausnitzii treatment improves hepatic health and reduces adipose tissue inflammation in high-fat fed mice. ISME J..

[86] Maioli T.U., Borras-Nogues E., Torres L., Barbosa S.C., Martins V.D., Langella P., Azevedo V.A., Chatel J.M. (2021). Possible Benefits of *Faecalibacterium prausnitzii* for Obesity-Associated Gut Disorders. Front. Pharmacol..

[87] Yang M., Wang J.H., Shin J.H., Lee D., Lee S.N., Seo J.G., Shin J.H., Nam Y.D., Kim H., Sun X. (2023). Pharmaceutical efficacy of novel human-origin *Faecalibacterium prausnitzii* strains on high-fat-diet-induced obesity and associated metabolic disorders in mice. Front. Endocrinol..

[88] Everard A., Belzer C., Geurts L., Ouwerkerk J.P., Druart C., Bindels L.B., Guiot Y., Derrien M., Muccioli G.G., Delzenne N.M. (2013). Cross-talk between Akkermansia muciniphila and intestinal epithelium controls diet-induced obesity. Proc. Natl. Acad. Sci. USA.

[89] Depommier C., Everard A., Druart C., Plovier H., Van Hul M., Vieira-Silva S., Falony G., Raes J., Maiter D., Delzenne N.M. (2019). Supplementation with Akkermansia muciniphila in overweight and obese human volunteers: A proof-of-concept exploratory study. Nat. Med..

[90] Suenaert P., Segers A., Rymenans L., Devroye H., Moll J.M., Cani P.D., de Vos W.M. (2026). Effect of pasteurized *Akkermansia muciniphila* MucT on insulin sensitivity, body composition, and GLP-1 production in subjects with metabolic syndrome: Impact of low baseline gut *Akkermansia* levels. Gut Microbes.

[91] Tolhurst G., Heffron H., Lam Y.S., Parker H.E., Habib A.M., Diakogiannaki E., Cameron J., Grosse J., Reimann F., Gribble F.M. (2012). Short-chain fatty acids stimulate glucagon-like peptide-1 secretion via the G-protein-coupled receptor FFAR2. Diabetes.

[92] Psichas A., Sleeth M.L., Murphy K.G., Brooks L., Bewick G.A., Hanyaloglu A.C., Ghatei M.A., Bloom S.R., Frost G. (2015). The short chain fatty acid propionate stimulates GLP-1 and PYY secretion via free fatty acid receptor 2 in rodents. Int. J. Obes..

[93] Cheng D., Xu J.-H., Li J.-Y., Wang S.-Y., Wu T.-F., Chen Q.-K., Yu T. (2018). Butyrate ameliorated-NLRC3 protects the intestinal barrier in a GPR43-dependent manner. Exp. Cell Res..

[94] Matheus V.A., Oliveira R.B., Maschio D.A., Tada S.F.S., Soares G.M., Mousovich-Neto F., Costa R.G., Mori M.A., Barbosa H.C.L., Collares-Buzato C.B. (2023). Butyrate restores the fat/lean mass ratio balance and energy metabolism and reinforces the tight junction-mediated intestinal epithelial barrier in prediabetic mice independently of its anti-inflammatory and epigenetic actions. J. Nutr. Biochem..

[95] Lu Y., Fan C., Li P., Lu Y., Chang X., Qi K. (2016). Short Chain Fatty Acids Prevent High-fat-diet-induced Obesity in Mice by Regulating G Protein-coupled Receptors and Gut Microbiota. Sci. Rep..

[96] Ge H., Li X., Weiszmann J., Wang P., Baribault H., Chen J.L., Tian H., Li Y. (2008). Activation of G protein-coupled receptor 43 in adipocytes leads to inhibition of lipolysis and suppression of plasma free fatty acids. Endocrinology.

[97] Kimura I., Ozawa K., Inoue D., Imamura T., Kimura K., Maeda T., Terasawa K., Kashihara D., Hirano K., Tani T. (2013). The gut microbiota suppresses insulin-mediated fat accumulation via the short-chain fatty acid receptor GPR43. Nat. Commun..

[98] De Vadder F., Kovatcheva-Datchary P., Goncalves D., Vinera J., Zitoun C., Duchampt A., Bäckhed F., Mithieux G. (2014). Microbiota-generated metabolites promote metabolic benefits via gut-brain neural circuits. Cell.

[99] Kelly C.J., Zheng L., Campbell E.L., Saeedi B., Scholz C.C., Bayless A.J., Wilson K.E., Glover L.E., Kominsky D.J., Magnuson A. (2015). Crosstalk between Microbiota-Derived Short-Chain Fatty Acids and Intestinal Epithelial HIF Augments Tissue Barrier Function. Cell Host Microbe.

[100] Yan H., Ajuwon K.M. (2017). Butyrate modifies intestinal barrier function in IPEC-J2 cells through a selective upregulation of tight junction proteins and activation of the Akt signaling pathway. PLoS ONE.

[101] Asher G., Sassone-Corsi P. (2015). Time for food: The intimate interplay between nutrition, metabolism, and the circadian clock. Cell.

[102] Franzago M., Alessandrelli E., Notarangelo S., Stuppia L., Vitacolonna E. (2023). Chrono-Nutrition: Circadian Rhythm and Personalized Nutrition. Int. J. Mol. Sci..

[103] Konstantinidou V., Jamshed H. (2024). Editorial: Chrononutrition and health. Front. Nutr..

[104] van der Merwe C., Münch M., Kruger R. (2022). Chronotype Differences in Body Composition, Dietary Intake and Eating Behavior Outcomes: A Scoping Systematic Review. Adv. Nutr..

[105] Horne J.A., Östberg O. (1976). A self-assessment questionnaire to determine morningness-eveningness in human circadian rhythms. Int. J. Chronobiol..

[106] Sato T., Sassone-Corsi P. (2022). Nutrition, metabolism, and epigenetics: Pathways of circadian reprogramming. EMBO Rep..

[107] Lamia K.A., Sachdeva U.M., DiTacchio L., Williams E.C., Alvarez J.G., Egan D.F., Vasquez D.S., Juguilon H., Panda S., Shaw R.J. (2009). AMPK regulates the circadian clock by cryptochrome phosphorylation and degradation. Science.

[108] Sato T., Sato S. (2023). Circadian Regulation of Metabolism: Commitment to Health and Diseases. Endocrinology.

[109] Hawley J.A., Sassone-Corsi P., Zierath J.R. (2020). Chrono-nutrition for the prevention and treatment of obesity and type 2 diabetes: From mice to men. Diabetologia.

[110] Paukkonen I., Törrönen E.-N., Lok J., Schwab U., El-Nezami H. (2024). The impact of intermittent fasting on gut microbiota: A systematic review of human studies. Front. Nutr..

[111] Verhoog S., Taneri P.E., Roa Díaz Z.M., Marques-Vidal P., Troup J.P., Bally L., Franco O.H., Glisic M., Muka T. (2019). Dietary Factors and Modulation of Bacteria Strains of *Akkermansia muciniphila* and *Faecalibacterium prausnitzii*: A Systematic Review. Nutrients.

[112] Effendi R.M.R.A., Anshory M., Kalim H., Dwiyana R.F., Suwarsa O., Pardo L.M., Nijsten T.E.C., Thio H.B. (2022). *Akkermansia muciniphila* and *Faecalibacterium prausnitzii* in Immune-Related Diseases. Microorganisms.

[113] de Cabo R., Mattson M.P. (2019). Effects of Intermittent Fasting on Health, Aging, and Disease. NEJM.

[114] Harvie M., Howell A. (2017). Potential Benefits and Harms of Intermittent Energy Restriction and Intermittent Fasting Amongst Obese, Overweight and Normal Weight Subjects—A Narrative Review of Human and Animal Evidence. Behav. Sci..

[115] Mattson M.P., Longo V.D., Harvie M. (2017). Impact of intermittent fasting on health and disease processes. Ageing Res. Rev..

[116] Tripathi A., Debelius J., Brennan C.A., Loomba R., Schnabl B., Knight R. (2018). The gut-liver axis and the intersection with the microbiome. Nat. Rev. Gastroenterol. Hepatol..

